# Microglia-derived CXCL2 induced neuronal ferroptosis via CXCR2/Jun axis in sepsis-associated encephalopathy

**DOI:** 10.3389/fimmu.2024.1512300

**Published:** 2025-03-06

**Authors:** Yu-Shen Yang, Jin-Wei Liang, Meng-Qin Pei, Yu-Ming Fang, Zhen-Dong Sun, He-Fan He

**Affiliations:** Department of Anesthesiology, The Second Affiliated Hospital of Fujian Medical University, Quanzhou, China

**Keywords:** sepsis-associated encephalopathy, neuronal ferroptosis, microglia activation, CXCL2/CXCR2 axis, crosstalk

## Abstract

**Background:**

Neuronal ferroptosis is a characteristic pathological change of sepsis-associated encephalopathy (SAE), which can be induced by activated microglia. CXCL2 is mainly secreted by inflammatory cells (neutrophil and microglia) and involved in neuronal damage. However, the specific mechanism behind microglia-neuron crosstalk in SAE remains unclear.

**Method:**

This study is to explore in which way microglia-secreted CXCL2 induced neuronal ferroptosis. For this purpose, the present study used CXCL2 knockdown (KD) mice to generate SAE model and determined effects of CXCL2 on neuronal ferroptosis. Afterward, BV2 and HT22 were used to instead of microglia and neuron respectively and the co-cultured system was used to simulate their interaction *in vivo* environment. RNA-sequencing technology was applied to investigate the key mechanism and targets of CXCL2-induced neuronal ferroptosis. siRNA was used to evaluate the function of key molecules.

**Results:**

Cecum ligation perforation (CLP) induced an obvious cognitive dysfunction, shorten the survival time and promoted the activation of microglia and neuronal loss. The level of inflammatory cytokines, ferroptosis-related markers and malonaldehyde was obviously lower and the level of glutathione was significantly higher in CXCL2 KD mice when compared with wide-type SAE mice. RNA-seq revealed that Jun is a potential target of CXCL2. The following experiments further demonstrated that microglia-secreted CXCL2 induced the neuronal ferroptosis, but siRNA-Jun in neuron can abolish this effect. In addition, siRNA-CXCL2 of microglia mitigated the neuronal ferroptosis induced by sepsis, while Jun agonist reversed this protective effect.

**Conclusion:**

In conclusion, microglia-derived CXCL2 could induce the occurrence of neuronal ferroptosis by targeting Jun. Thus, regulating the expression and secretion of CXCL2 will probably be a crucially novel strategy for the treatment of SAE.

## Background

Sepsis, a fatal organ dysfunction, is caused by a maladjusted host response to infection (1). According to statistics in 2017, there were approximate 48.9 million sepsis cases and 11.0 million deaths, accounting for 19.7% of all deaths worldwide (1). Sepsis-associated encephalopathy (SAE) is a common complication of sepsis, as characterized by confusion or even coma (2). Over 70% of sepsis patients are diagnosed with SAE, which is regarded as the most common reason of encephalopathy in intensive care unit (3). Unfortunately, sepsis-induced neurological dysfunction usually last for several years in many survivors and badly influence their quality of life, leading to a great social and economic burden. Although data on pathological mechanism contributing to SAE are scarce, several potentially factors have been determined, including blood–brain barrier (BBB) destruction, microglial activation and neuronal ferroptosis (4–6).

As the predominant immune cells in central nervous system (CNS), microglia have been demonstrated to exert a crucial role in the pathophysiology of SAE (7, 8). During sepsis, after periphery cytokines (IL-1β, IL-6 and TNF-α) enter into CNS via BBB, microglia are rapidly activated, displaying obviously functional changes and secreting a large number of aforementioned cytokines, which in turn further expands the neuroinflammatory response (9, 10). On the other hand, ferroptosis is a novel type of programmed cellular death, involving the accumulation of reactive oxygen species (ROS) caused by an increase in oxidative damage and intracellular iron ions induced by lipid peroxidation. Previous literatures have demonstrated the correlation between neuronal ferroptosis and SAE. For example, Chu et al. (11) demonstrated that sepsis induced the occurrence of neuronal ferroptosis and acetaminophen can reverse this process via the GPX4 signaling pathway. Xie et al (12) identified the association of neuronal ferroptosis with glutamate-mediated excitotoxic neuronal damage. Although these reports revealed the crucial role of neuron ferroptosis in SAE, they did not further explore the underlying mechanisms and primary “culprits”. Recently, several literatures have indicated that microglial activation can facilitate neuronal ferroptosis in certain neurological disease, such as Alzheimer’s disease (13). Thus, it is necessary to investigate whether microglial activated by sepsis promotes the progression of SAE via inducing neuronal ferroptosis and reveal the potential mechanism.

C-X-C motif chemokine ligand 2 (CXCL2), a member of CXC subfamily, is involved in inflammatory and immunoregulatory processes via targeting C-X-C motif chemokine receptor 2 (CXCR2). Recently, it has been demonstrated that overexpression of PDCD10 in glioblastoma recruits and activates microglia/macrophages via CXCL2 (14); in turn, activated BV-2 cells by ATP can also secrete and release CXCL2 (15). Importantly, previous studies have confirmed that CXCL2 can regulate the activity and axis growth of neuron. For instance, Teona Deftu et al. (16) indicated that CXCL2 stimulus significantly blocked the axon outgrowth, while blocking CXCR2 can reverse these effects. In addition, the short-term incubation of CXCL2 can effectively reduce TRPV1 desensitization in TRPV1^+^/IB4^+^ dorsal root ganglia neurons (17). Interestingly, CXCL2 overexpression was also found to induce the occurrence of ferroptosis in cancer cells. However, whether sepsis-activated microglia can secrete CXCL2 and thus promote the neuronal ferroptosis remains unclear.

This study is to explore the role and underlying mechanism of microglia-neuron crosstalk in SAE and determine whether activated microglia promote neuronal ferroptosis via secreting CXCL2. Finally, the potential mechanism by which microglia-secreted CXCL2 induce neuronal ferroptosis will be further investigated.

## Material and methods

All experimental procedures were performed in accordance with the Guide for the Care and Use of Laboratory Animals (National Institutes of Health, Bethesda, MD, USA) and approved by the Second Affiliated Hospital of Fujian Medical University (approval number: 2023156).

### Establishment of SAE model

CXCL2 knockdown mice was purchased from Cyagen biosciences (Suzhou) lnc. A SAE model was prepared using the cecum ligation perforation (CLP) method. In summary, the mice were anaesthetized with 1% sodium pentobarbital. Following disinfection of the abdominal skin, a 1.5 cm incision was made in the midline of the abdomen to expose the cecum. The exposed cecum was then ligated with a 4-0 suture below the ileocecal valve, at a distance of 5 mm from the tip of the cecum. Subsequently, the cecum was repositioned within the abdominal cavity, with a minimal quantity of feces extruded through a permeable opening created with a 21-gauge needle. The incision was then sutured. Subcutaneous injection of 1 ml of saline was administered to all mice for resuscitation purposes. Following resuscitation, the mice were returned to their original cages. The mice in the sham-operated group underwent the same incision in the abdominal cavity as the other groups, but without ligation or perforation. All mice were permitted to eat and drink ad libitum, with automatic temperature control at 22 ± 2°C, humidity of 55-65%, and a light-dark cycle of 12 h - 12 h. Four to five mice were housed in each cage.

### Barnes maze

The Barnes maze experiments were conducted on a circular platform with a diameter of 90 cm and a height of 90 cm above the ground. The mice were positioned at the center of a circular platform (SD Instruments, San Diego, CA) comprising 20 equally spaced holes. One of the holes was connected to a dark chamber, designated as the “target box.” The mice were stimulated to search for the target box using an obnoxious noise of 85 dB and bright light of 200 W. At the outset of each trial, the animals were subjected to a habituation phase in a start box situated at the center of the maze. After a 5s interval, the start box was removed, and the mouse was permitted to explore the area for 300 s. The entire procedure encompassed four training days, with each day comprising four trials of 5 min each, and a single test day. The behavioral responses were recorded and evaluated using a video tracking system (SD Instruments, San Diego, CA).

### RNA extraction and RT-qPCR

Total RNA was extracted from cultured HT22 neurons and BV2 microglia using a Trizol kit (Thermo Fisher Scientific). cDNA was synthesized using a cDNA synthesis kit (Thermo Fisher Scientific) in accordance with the manufacturer’s instructions, followed by RT-qPCR reactions conducted on a StepOnePlus Real-Time PCR instrument (Applied Biosystems, Foster City, CA, USA). The RT-qPCR reaction conditions were as follows: an initial denaturation at 94°C for 3 min, followed by 45 s of denaturation at 94°C, 30 s of annealing at 56°C, and 45 s of extension at 72°C, for a total of 45 cycles. The relative quantitative 2^-ΔΔCt^ method was employed to ascertain the expression level of the genes, with glyceraldehyde-3-phosphate dehydrogenase (GAPDH) serving as the housekeeping gene. The PCR primer sequences utilized are provided in **Supplementary Table 1**.

### Western blot

The total protein was lysed with RIPA protein extract, which contains a mixture of protease and phosphatase inhibitors, and the protein concentration was measured using a BCA kit (Beyotime Institute of Biotechnology, Inc., Shanghai, China) in accordance with the manufacturer’s instructions. The samples were separated by SDS-PAGE to ensure equal amounts of protein (20 μg per lane) and transferred to polyvinylidene difluoride (PVDF) membranes (Thermo Fisher Scientific). The membranes were sealed with 5% skimmed milk at room temperature for 1h. Subsequently, the membranes were incubated with the primary antibody TFRC (1:1000, A21622, ABclonal), PTGS2 (1:1000, CY5580, Abways), Jun (1:1000, 380397, Zenbio), and GAPDH (1:10000, ab181602, abcam) at 4°C overnight, followed by incubation with the anti-rabbit IgG secondary antibody at room temperature for 1h. Thereafter, the blots were developed using ECL reagent and the blots were imaged. Each experiment was repeated three times, and the mean values were calculated.

### Immunofluorescence

Brain cryosections or treated cells were permeabilized with 0.1% Triton X-100 (Sigma-Aldrich) for a period of 10 min at RT. Subsequently, the fixed tissues and cells were incubated with 3% bovine serum albumin (Sigma-Aldrich) for 30 min at 37°C. Next, the brain sections or cells were incubated with the primary antibodies overnight at 4°C. Following a rinse with PBS, tissues and cells were incubated with the corresponding secondary antibody at RT in the dark for one hour. Nuclear counterstaining with DAPI was thereafter performed. The samples were imaged under a fluorescence microscope (Nikon, Tokyo, Japan), and 15 fields of view (5 fields of view/slice × 3 slices/mouse) were randomly selected for the quantification of positive cells using ImageJ software.

### Nissl staining

A Nissl staining assay (methylene blue method) was used to evaluate the hippocampal neuronal damage. All of procedures were performed according to the instructions of commercial kit (Cat.No, S1096; Bioss, Beijing, China). Briefly, the slices were dewaxed and hydrated, followed by staining with Methylene Blue Stain for 10 min. Subsequently, the sections were differentiated with Nissl Differentiation fluid for 5-10s and then treated by Ammonium Molybdate Solution for 5 min. Finally, the sections were dehydrated with 95% and 100% ethanol, and fixed with Neutral balsam. Images were captured using a light microscope (Nikon, Japan).

### Cell culture (HT22 and BV2 cells)

The BV2 cell line was purchased from the Shanghai Zhong Qiao Xin Zhou Biotechnology Co., Ltd. (Shanghai, China), while the HT22 mouse hippocampal neurons were sourced from Thermo Fisher Scientific Inc. (Waltham, MA, USA). BV2 microglia and HT22 cells were cultured in DMEM (Gibco; Thermo Fisher Scientific, Inc.) supplemented with 10% fetal bovine serum (FBS) (Gibco; Thermo Fisher Scientific, Inc.) and 1% penicillin/streptomycin at 37°C and 5% CO_2_ atmosphere for incubation.

### siRNA transfection

BV2 microglia or HT22 neurons were plated in 6-well plates and grown to 50–60% confluency in medium containing 10% fetal bovine serum without 1% penicillin/streptomycin. Subsequently, the cells were transfected with siRNA targeting CXCL2 or Jun using the siRNA reagent system (HyCyte Biotechnology, Suzhou, China) according to the manufacturer’ s protocol, with scrambled siRNAs (HyCyte Biotechnology, Suzhou, China) serving as a negative control.

### CXCL2, malondialdehyde and glutathione measurement

A suspension of cells (4 ×10^5^ cells per well) was introduced into 6-well plates and incubated at 37°C overnight. The cell supernatants were aspirated into 1.5ml EP tubes. The levels of MDA were evaluated using commercial kits following the manufacturer’s instructions (Cat.No, BC0025; Beijing Solarbio Science & Technology Co., Ltd, Beijing, China). Then, absorbance was tested at 532 nm and 600 nm. Additionally, the levels of GSH were evaluated with Glutathione Assay Kit (Cat.No, A006-2-1; Nanjing Jiancheng Bioengineering Research Institute, Naijing, China) according to the manufacturer’s instructions, and the content of GSH was determined via calculating the absorbance values at 405 nm. CXCL2 in mice serum and medium supernatant of BV2 microglia were detected using the ELISA system (R&D systems, MN).

### FerroOrange staining

Fe^2+^ levels were assessed using FerroOrange staining kit (Cat.No, F374; dojingo, Shanghai, China). HT22 cells were seeded in a confocal dish at 1×10^5^ cells/ml density and cultured for 24 h. Based on the specific requirement, the cells were next treated for 24 h. Subsequently, a working solution of 1 μmol/l FerroOrange fluorescent probe was added to the cells. The cells were maintained at 37°C and 5% CO_2_ atmosphere for incubation for 30 min. Finally, images were obtained using confocal microscopy (Nikon, Japan).

### RNA-sequence analysis

For the purpose of RNA-seq analysis, the HT22 cells were treated with rmCXCL2 for 24 h. Whole genome RNA was extracted using the TRIZOL method, and the RNA concentration was quantified using a NanoDrop 2000 (Thermo Fisher Scientific). The RNA was then purified using the QIAgen RNEasy kit. Sequencing libraries were constructed using the NEBNext Ultra RNA Library Preparation Kit from NEB (USA), and the library quality was assessed using the Agilent Bioanalyzer 2100 system. The resulting samples were then analyzed on two channels using an Illumina 2000 Hi-seq machine, resulting in the generation of paired-end reads.

### Statistics

All data were subjected to statistical analysis using SPSS Statistics 26 software, and graphs were generated using GraphPad Prism 9 software (GraphPad Software Inc., San Diego, CA, USA). One-way analysis of variance (ANOVA) and Tukey’s *post hoc* test were employed to perform statistical analyses. Additionally, an unpaired t-test was used to compare the difference between two groups. A p-value of less than 0.05 was considered statistically significant.

## Results

### SAE model stability

**Figure 1:**
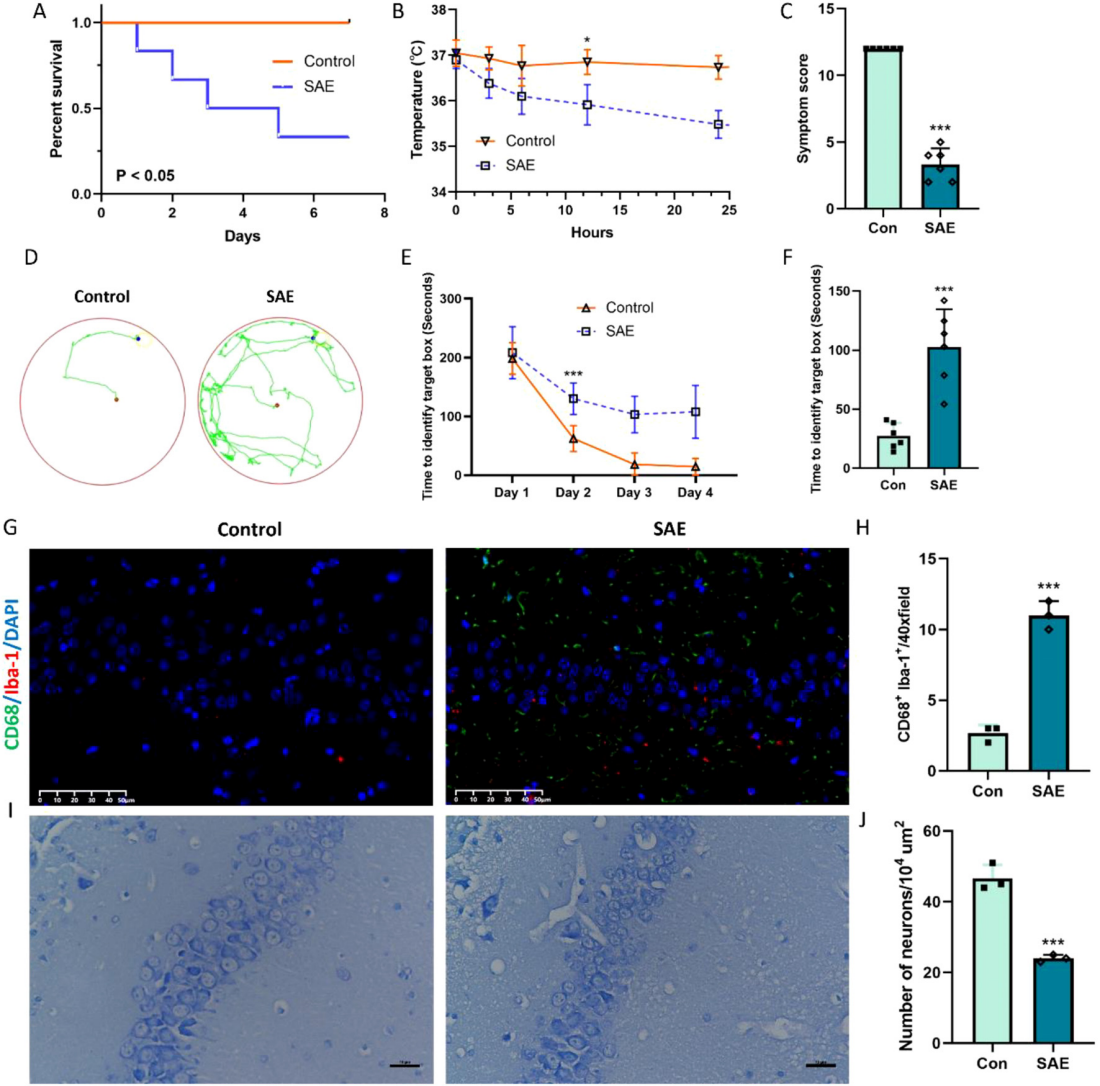
Representative change after CLP model. **(A)** Percentage of surviving animals each group 7 days after CLP (n=6); **(B)** Body temperature in each group 24 h after CLP (n=6); **(C)** Symptom score (n=6); **(D-F)** Barns maze (n=6); **(G, H)** Representative immunofluorescence images of microglial activation and the quantification of CD68/Iba-1-positive cells in the hippocampus (n=3). Scale bar, 50 µm; **(I, J)** Nissl-stained hippocampal sections indicating neuronal loss with quantification representing average number of Nissl positive cells (n=3). Scale bar, 10 µm. *p<0.05, ***p<0.001, vs. Con.

In order to closely mimic the progression and characteristics of human sepsis and SAE, we used the most quintessential CLP sepsis model and evaluated the survival status, body temperature, symptom score, and cognitive change of SAE mice after surgery. The mortality rate of mice in the CLP group is 67%, which was obviously lower survival rate than the sham mice (**Figure 1A**, p < 0.05). In addition, SAE mice displayed noticeably lower body temperature and severer symptom score when compared with sham mice (**Figures 1B, C**, p < 0.05). Subsequently, Barrns maze was performed to evaluate the cognitive change of SAE mice. After being trained for 4 days, the cognitive function of mice in both groups to enter in the target hole was obviously improved in the training period (**Figures 1D, E**, p < 0.05). Meanwhile, 7 days after surgery, the spatial learning ability of mice in SAE group was markedly impaired when compared with the mice in control group (**Figure 1F**, p < 0.05). To further depict the changes of hippocampus tissue at the cellular level (mainly microglia and neuron) after CLP surgery, CD68/Iba-1 staining and Nissl staining were performed to respectively evaluate the microglia activity and neuron death. Preliminary experiments displayed that the number of CD68/Iba-1 positive microglia obviously increased in SAE group (**Figures 1G, H**, p < 0.05); while neurons in hippocampal CA1 layers showed a significant decrease after treatment with CLP (**Figures 1I, J**, p < 0.05). All these changes in mice treated by CLP are consistent with the characteristic features of SAE, demonstrating the successful establishment of SAE mice.

### CXCL2 expression was upregulated in the hippocampus tissue and microglia after sepsis/LPS stimulus

**Figure 2:**
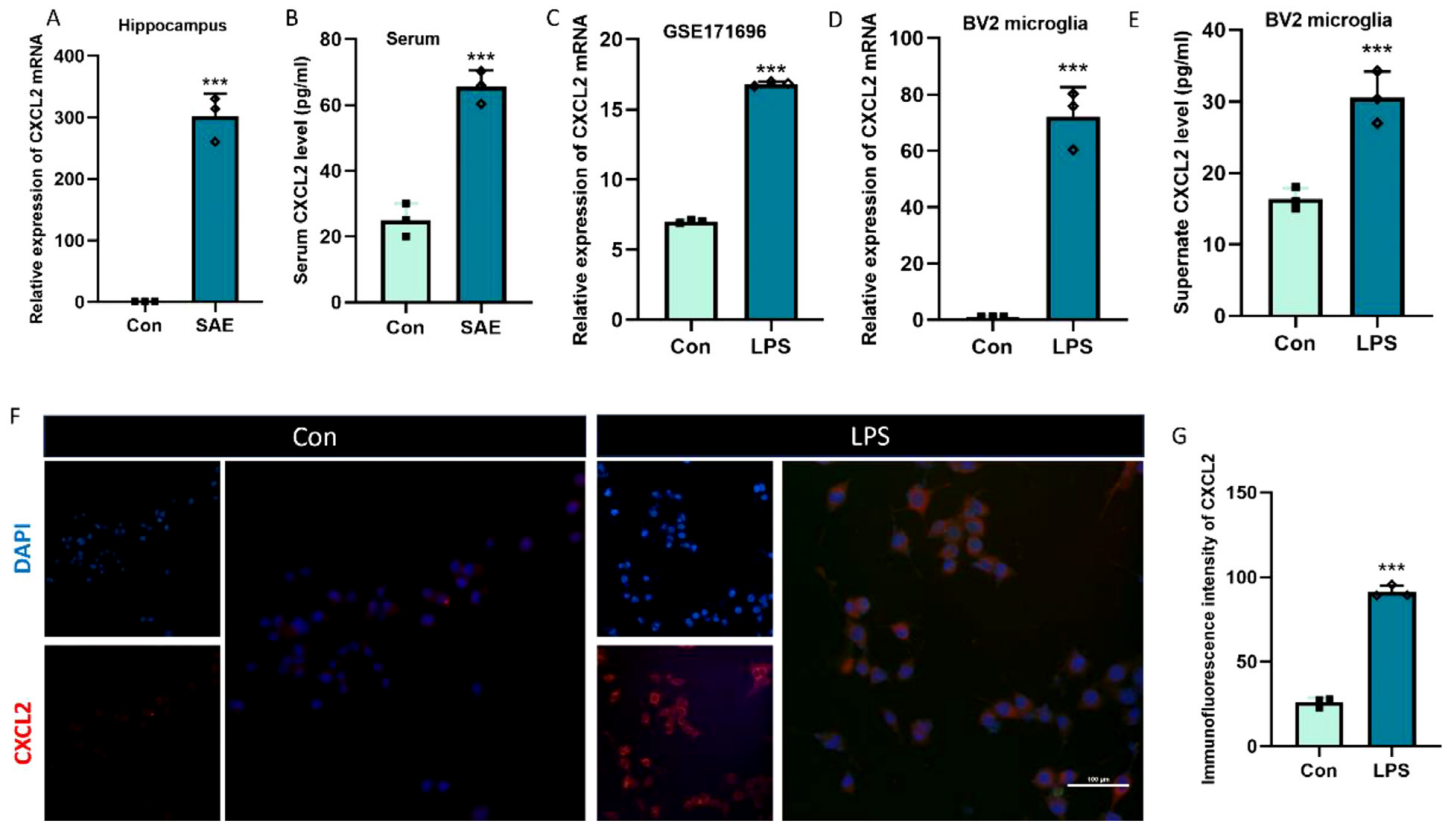
The expression of CXCL2 in hippocampus tissue and microglia after sepsis/LPS stimulus. **(A)** RT-qPCR results showing the levels of CXCL2 in hippocampus tissue; **(B)** ELISA results showing the levels of CXCL2 in mice serum; **(C)** The results showing the levels of CXCL2 in BV2 microglia from GSE171696 dataset; **(D)** RT-qPCR results showing the levels of CXCL2 in BV2 microglia; **(E)** ELISA results showing the levels of CXCL2 in BV2 microglia supernate; **(F, G)** Immunofluorescence results showing the levels of CXCL2 in BV2 microglia. Scale bar, 10 µm. n=3, ***p<0.001, vs. Con.

We detected the CXCL2 expression in the hippocampus 24 h after CLP at first. RT-qPCR and Elisa results demonstrated that the expression of CXCL2 mRNA and protein was obviously upregulated in SAE group, when compared with Con group (**Figures 2A, B**, p < 0.05). It has been reported that CXCL2 can be secreted by several inflammatory cells, including neutrophils, macrophages, astrocytes, or microglia (18). In CNS, microglia are the predominant immune cells. Thus, we next evaluated the CXCL2 expression in the BV2 microglia after LPS stimulus. The GSE171696 dataset, aiming at developing gene expression signatures for BV2 microglia activation, was firstly used to evaluate the expression difference of CXCL2 in BV2 microglia before and after activation. We found that the mRNA level of CXCL2 was significantly elevated in LPS-activated microglia compared with PBS-treated one (**Figure 2C**, p < 0.05). Subsequently, we performed a series of *in vitro* experiments to fully verify the aforementioned change of CXCL2 expression in microglia. The results from RT-qPCR, Elisa, and immunofluorescence further demonstrated the points that activated microglia by LPS displayed markedly higher expression level of CXCL2 in comparison with resting microglia (**Figures 2D-G**, p < 0.05).

### CXCL2 knockdown alleviates sepsis-induced neuronal ferroptosis

**Figure 3:**
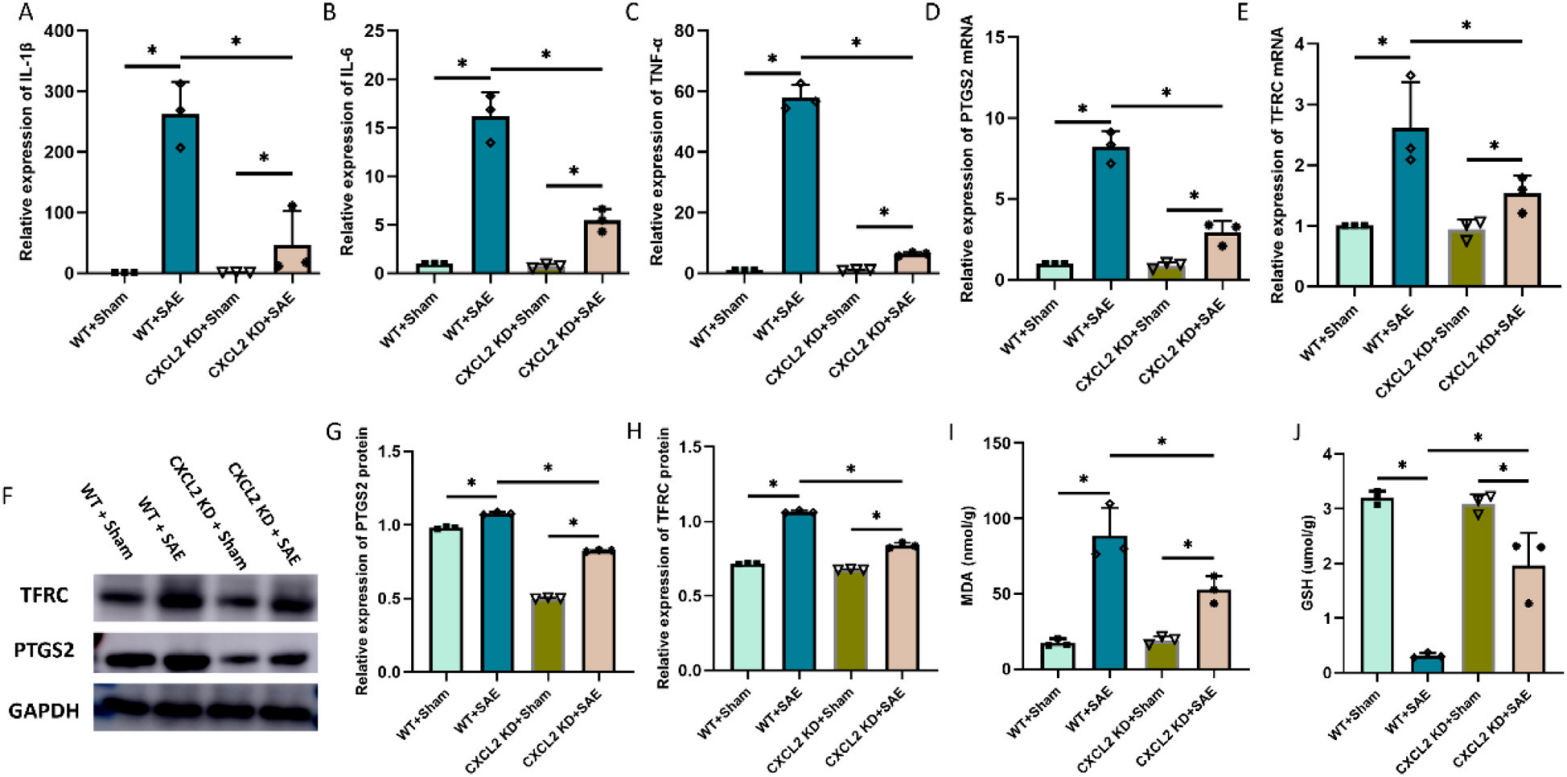
Effects of CXCL2 on neuronal ferroptosis and neuroinflammatory responses in sepsis associated encephalopathy (SAE) model. **(A-C)** RT-qPCR results showing the levels of inflammatory cytokines in hippocampus tissue; **(D, E)** RT-qPCR results showing the levels of ferroptosis-related markers (PTGS2 and TFRC) in hippocampus tissue; **(F-H)** Western blot results showing the levels of ferroptosis-related markers (PTGS2 and TFRC) in hippocampus tissue; **(I, J)** Quantitative analysis of GSH and MDA. n=3, *p<0.05.

CXCL2 was found to induce the occurrence of ferroptosis in cancer cell. To investigate the role of CXCL2 in neuronal ferroptosis induced by sepsis, CXCL2 knockdown mice were subjected to CLP. As expected, the expression of proinflammatory cytokines in hippocampus tissue, including IL-1β, IL-6, and TNF-α, in CXCL2 knockdown septic mice was strikingly lower than that in wide type septic mice (**Figures 3A-C**, p < 0.05). Subsequently, CXCL2 knockdown was found to alleviate CLP induced neurons ferroptosis, as evidenced by higher GSH level, and lower ferroptosis-related markers expression (PTGS2 and TFRC) and MDA level (**Figures 3D-J**, p < 0.05).

### Jun is a potential downstream target of CXCL2/CXCR2 in microglia-neuron crosstalk

**Figure 4:**
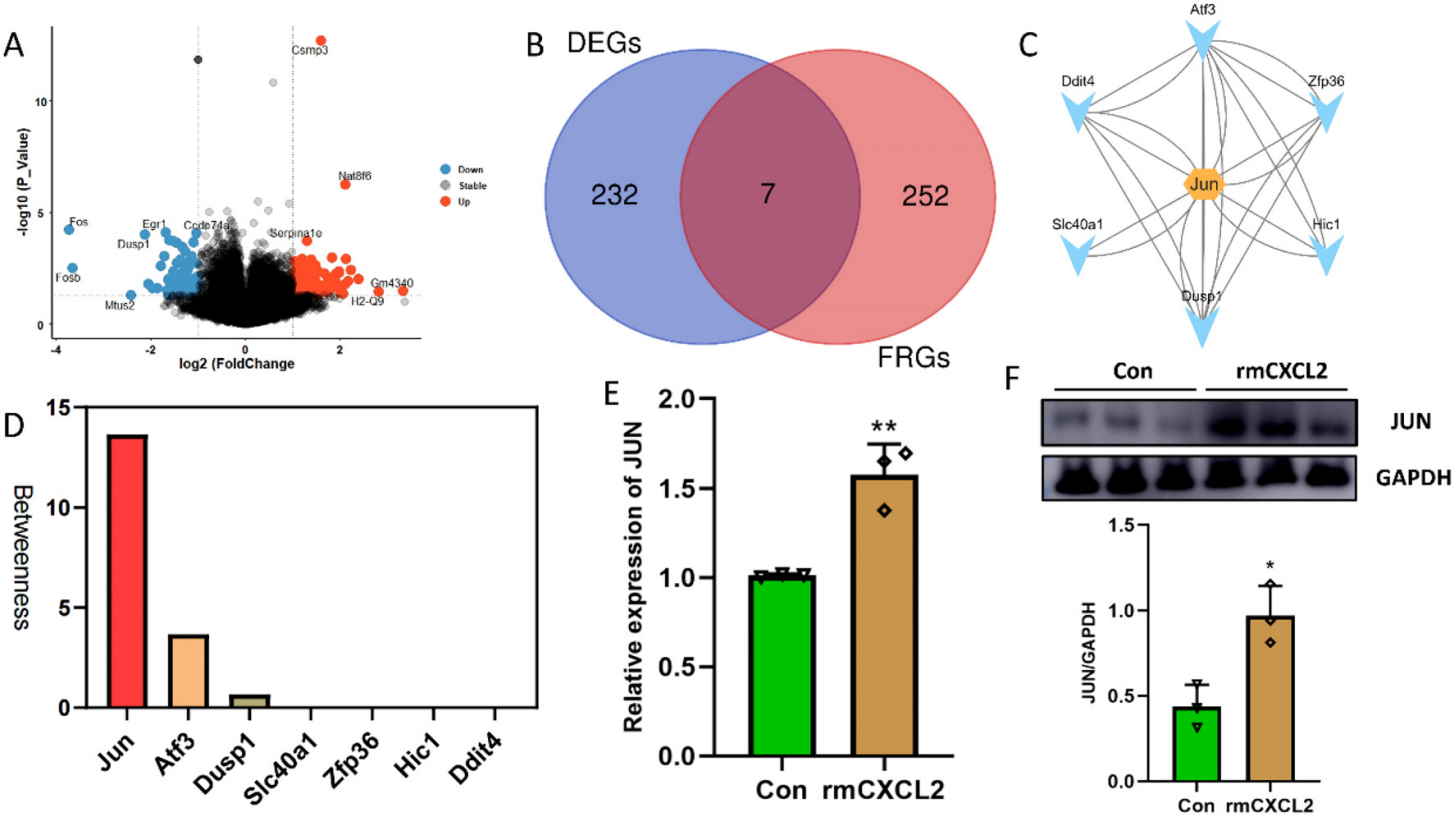
RNA-sequence analysis of HT22 neurons after rmCXCL2 stimulus. **(A)** Volcano plot displays differentially expressed genes (DEGs) of rmCXCL2-treated HT22 neurons compared with Con group. **(B)** Venn plot interesting DEGs with ferroptosis-related genes (FRGs). **(C)** Protein-protein interaction of hub FRGs; **(D)** The results from CytoHubba plug calculating the Betweenness value of hub FRGs; **(E)** RT-qPCR results showing the levels of Jun in HT22 neurons; **(F)** Western blot results showing the levels of Jun in HT22 neurons. n=3, *p<0.05, **p<0.01, vs. Con.

To investigate the potential mechanism through which CXCL2 induces neuronal ferroptosis, RNA-seq analysis was conducted to determine the expression profile of HT22 hippocampal neurons following rmCXCL2 treatment. Compared with the neurons treated by PBS, a total of 239 differentially expressed genes (DEGs) were demonstrated in the samples treated by rmCXLC2, which contained 84 downregulated and 155 upregulated genes (**Figure 4A**). We then intersected these DEGs and ferroptosis-related genes (FRGs) to obtain seven hub FRGs (hFRGs) (**Figure 4B**). **Figure 4C** displayed the underlying connection among the hFRGs via PPI network. The CytoHubba plug was then utilized to calculated the betweenness value of hFRGs and Jun was found to be the top 1 key target with the highest score (**Figure 4D**). Subsequently, we performed several *in vitro* experiments to validate the expression change of Jun in HT22 hippocampal neuron before and after rmCXCL2 treatment. The results indicated that rmCXCL2 obviously upregulated the expression of Jun mRNA and protein (**Figures 4E, F**, p < 0.05). We further evaluated the effect of CXCL2 on Jun expression via *in vivo* experiments and found that CXCL2 knockdown could effectively downregulated the Jun expression at mRNA and protein level (**Supplementary Figure 1**, p < 0.05). These clues hinted that microglia-activated CXCL2/CXCR2 axis probably causes the neuronal ferroptosis via targeting Jun.

### CXCL2/CXCR2 axis causes the neuronal ferroptosis via targeting Jun

**Figure 5:**
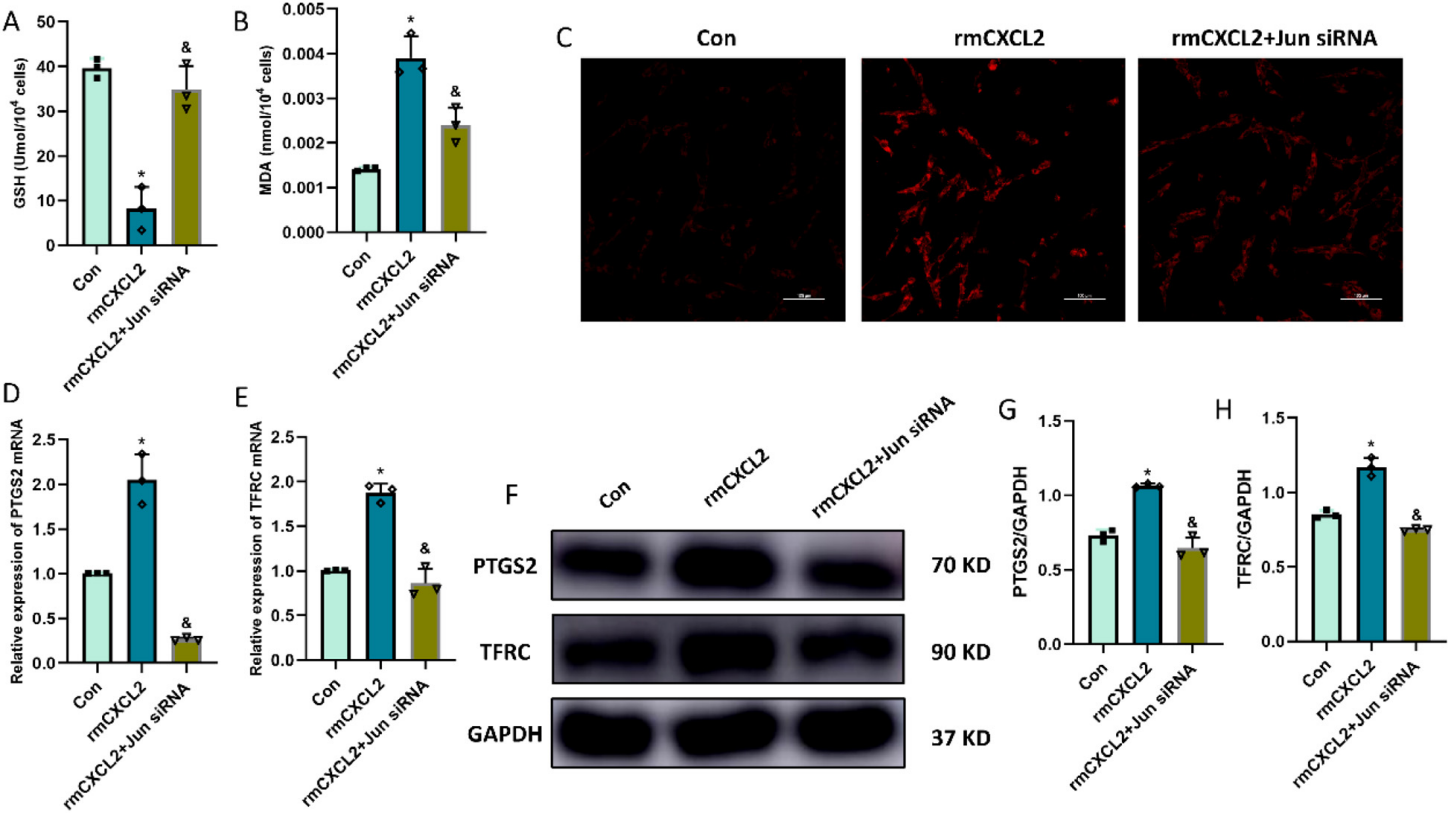
rmCXCL2 induced neuronal ferroptosis and activated Jun pathway in HT22 cells. **(A, B)** Quantitative analysis of GSH and MDA; **(C)** FerroOrange results showing the levels of Fe^2+^ in HT22 neurons. Scale bar, 100 µm; **(D, E)** RT-qPCR results showing the levels of ferroptosis-related markers (PTGS2 and TFRC) in HT22 neurons; **(F-H)** Western blot results showing the levels of ferroptosis-related markers in HT22 neurons. n=3, *p<0.05 vs. Con; ^&^p<0.05 vs. rmCXCL2 group.

**Figure 6:**
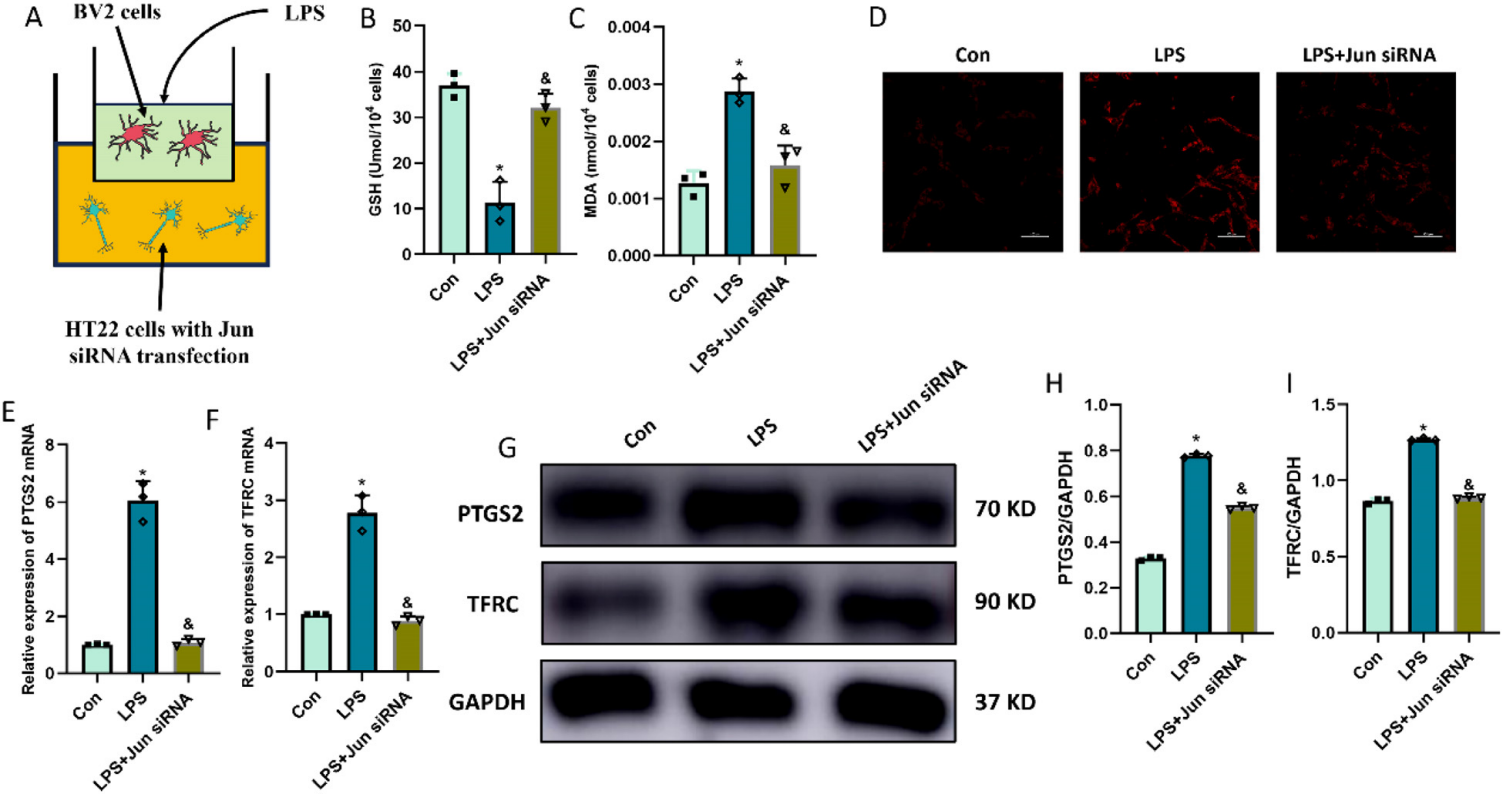
LPS-activated microglia induced neuronal ferroptosis in HT22 cells. **(A)** co-culture system of BV2 and HT22 cells; **(B, C)** Quantitative analysis of GSH and MDA; **(D)** FerroOrange results showing the levels of Fe^2+^ in HT22 neurons. Scale bar, 100 µm; **(E, F)** RT-qPCR results showing the levels of ferroptosis-related markers (PTGS2 and TFRC) in HT22 neurons; **(G-I)** Western blot results showing the levels of ferroptosis-related markers in HT22 neurons. n=3, *p<0.05 vs. Con; ^&^p<0.05 vs. LPS group.

In order to explore the function and effect of Jun in neuronal ferroptosis induced by microglia-secreted CXCL2, we transfected Jun siRNA to downregulate the expression of Jun in HT22 hippocampal neurons before rmCXCL2 treatment. The outcomes demonstrated that rmCXCL2-alone treatment promoted neuronal ferroptosis, as evidenced by the decreased GSH levels, elevated MDA levels, excessive iron uptake, and altered ferroptosis-related protein expression [specifically PTGS2 and TFRC]. However, when repressed Jun, the neuronal ferroptosis induced by rmCXCL2 was reversed compared to rmCXCL2‐alone group (p < 0.05; **Figures 5A-H**). Additionally, we established a microglia-neuron coculture system to explore whether microglia activation induced neuronal ferroptosis via Jun. HT-22 cells treated with Jun siRNA or medium were placed in the lower chamber, and BV-2 cells treated with LPS or medium were added to the upper chamber for 24 hours. The results suggested that LPS-activated BV2 microglia can induce the occurrence of neuronal ferroptosis, but Jun siRNA transfection into HT22 neuron cells could curtail the ferroptosis of neuron (p < 0.05; **Figures 6A-I**).

### Microglia-derived CXCL2 induces the neuronal ferroptosis via targeting Jun

**Figure 7:**
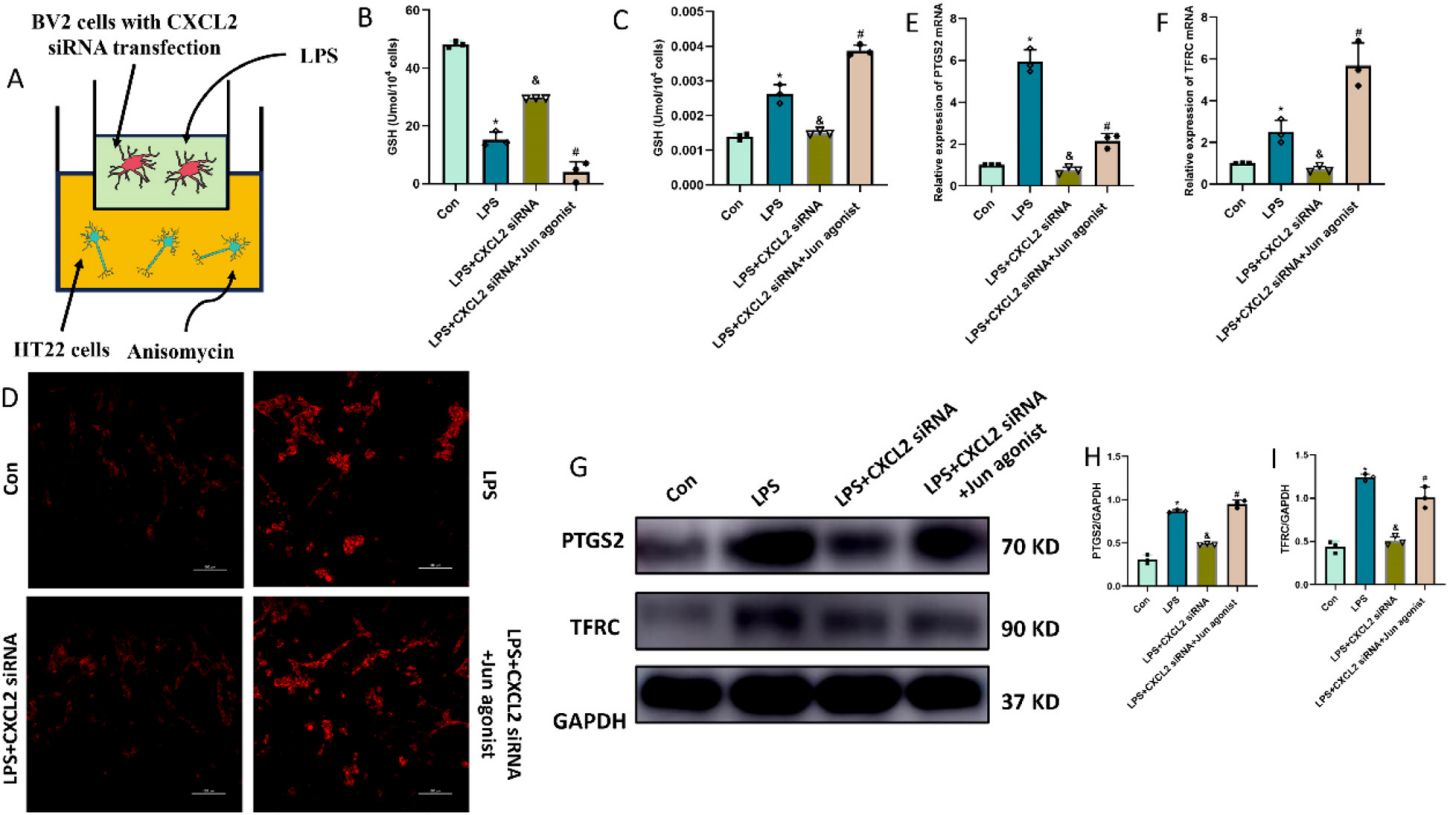
Microglia-secreted CXCL2 induced neuronal ferroptosis and activated Jun pathway in HT22 cells. **(A)** co-culture system of BV2 and HT22 cells; **(B, C)** Quantitative analysis of GSH and MDA; **(D)** FerroOrange results showing the levels of Fe^2+^ in HT22 neurons. Scale bar, 100 µm; **(E, F)** RT-qPCR results showing the levels of ferroptosis-related markers (PTGS2 and TFRC) in HT22 neurons; **(G-I)** Western blot results showing the levels of ferroptosis-related markers in HT22 neurons. n=3, *p<0.05 vs. Con; ^&^p<0.05 vs. LPS group; ^#^p<0.05 vs. LPS+CXCL2 siRNA group.

Finally, we questioned whether microglia-derived CXCL2 induces the neuronal ferroptosis via targeting Jun. In the microglia-neuron coculture system, thus, we added BV-2 cells treated with LPS+CXCL2 siRNA in the upper chamber, while we added HT-22 cells treated with Jun agonist or medium in the lower chamber for 24 h. As shown by **Figure 7**, LPS-alone treatment promoted neuronal ferroptosis, but CXCL2 knockdown can revere the effects. However, when Jun agonist was added, the inhibitory effect of CXCL2 knockdown on neuronal ferroptosis was obviously hindered compared to LPS+CXCL2 knockdown group (p < 0.05; **Figures 7A-I**).

## Discussion

It is a great truth that microglia are required for neural immunoregulation and the maintenance of neuronal homeostasis. To perform their functions, microglia, on the one hand, can sense neuronal activity via expressing various neurotransmitter receptors, whose activation triggers the key functions of microglia, including cellular motility, cytokines secretion and phagocytosis (19–21). On the other hand, they can also control the neuronal activity through activating a large variety of receptors expressed by neurons via releasing soluble molecules. Thus, the continuous microglia-neurons crosstalk contributes to the maintenance of neuronal activity and function. Undesirable activation of microglia probably impairs the normal communication with neurons, and in consequence memory, learning, and other cognitive functions (22).

Recently, increasing evidence have demonstrated the abnormal microglia-neuron communication in many brain diseases. For example, cytokines released by microglia, including IL-1β (23), IL6 (24), TNFα (25), are associated with the regulation of neuronal functions, contributing to detrimental effects. Liu et al. revealed that the activated microglia facilitate the activation of GRPR^+^ neurons via the NLRP3/caspase-1/IL-1β/IL1R1 axis, thus contributing to several different chronic itches (26). In addition, the reduction of microglia-neuron crosstalk caused by the decrease in CX3CR1/CX3CL1 axis leads to the homeostatic dysfunction, thus affecting physiological processes (27). Microglia activation could induce CCL2 expression and deletion of CCL2 from microglia facilitates VGluT1 synapse preservation and recovery (28). However, whether the altered crosstalk of microglia with neuron can be observed in SAE remains unclear. In this study, we reveal a previously unproven phenomenon and mechanism in which sepsis-activated microglia induces the occurrence of neuronal ferroptosis via the CXCL2/CXCR2/Jun axis.

Chemokines, an abbreviation of *chemotactic cytokines*, consist of a superfamily of small peptides (8-14 KD) that act via binding to G protein-coupled receptors. Its’ function involves the activation of leukocyte chemotaxis and immune responses under pathological infection, as well as the regulation of the occurrence and metastasis of tumors (29–31). The chemokine superfamily is classified into four subgroups according to the configuration of the N-terminal cysteine residues, including CXC, CC, C, and CX3C subfamilies (32). Different chemokines have different physio-pathologic functions and many of them are correlated with a variety of neurological diseases. For example, CXCL12 overexpression was found to promote neuroinflammation and cognitive dysfunction in neuropathic pain via mediating monocyte transmigration into brain perivascular space (33). Additionally, Zhu et al. indicated that CCL2 probably mediates the local sympathetic effects on neuropathic pain and thus regulates the regeneration process (34). The link of CXCL12 and CX3CL1 expression to human nerve sheath tumors has also been reported in recent literature (35). These findings highlight the important tole of chemokines in neurological disease, thus promoting them to become a hot area of research for neurologist and psychiatrist. However, among them, the literatures revealing the association of CXCL2 with various neurological diseases remain tiny.

On the other hand, as one of the chemokine family members, CXCL2 has crucial physiological functions and exerts a crucial role in the inflammation response. Many literatures have indicated that CXCL2 is involved in the progression and prognosis of sepsis. For example, Villar et al. found that CXCL2 gene with a tandem repeat polymorphism (AC)n at position -665 is an independent factor of mortality and related to better prognosis in patients with severe sepsis (35). Flores et al. demonstrated that CXCL2 gene variants may enhance the susceptibility to sepsis and thus facilitate the development of severe sepsis (36). Furthermore, a recent original study revealed that macrophagic extracellular vesicle CXCL2 can aggravate the progression of sepsis via recruiting and activating the neutrophil CXCR2/PKC/NOX4 axis (37). Nevertheless, the role of CXCL2 in SAE is still unclear. In the present study, we found CXCL2 expression was upregulated in the hippocampus tissue, blood serum, microglia and medium supernatant after sepsis/LPS stimulus. Importantly, we further revealed that microglia-secreted CXCL2 can induce the occurrence of neuronal ferroptosis in SAE.

In order to explore the mechanism by which CXCL2 leads to neuronal ferroptosis, RNA-seq analysis was constructed to depict the expression profile of HT22 hippocampal neuron after rmCXCL2 treatment. The results indicated that Jun is a potential downstream target of CXCL2/CXCR2 in microglia-neuron crosstalk. Jun, which is also known as activator protein-1 (AP-1) subunit, is the putative transforming gene of avian sarcoma virus 17 and encodes c-Jun protein [15]. The alteration of JNK/c-Jun signaling pathway in sepsis has been increasingly reported. Liu et al. found that the inhibition of the JNK/c-Jun signaling pathway by HIPK3 could potentially block the progression of sepsis (38). Furthermore, Kenzel et al. confirmed the importance of JNK/c-Jun signaling pathway in the transcriptional activation of proinflammatory cytokine genes responding to Group B streptococcus and further illustrated that the inhibition of JNK/c-Jun axis can retard GBS-induced cytokine formation and sepsis progression (39). On the other hand, Jun showed a close association with ferroptosis and iron metabolism. For example, Jun overexpression could alleviate erastin‐induced ferroptosis in Schwann cells by the upregulation of GPX4 and the downregulation of PTGS2 (40). However, in pancreatic cancer, Jun overexpression was found to upregulate the mRNA transcription and protein expression of TFRC by binding to its’ promoters, thus contributing iron metabolism (41). In this study, we also found that CLP-induced sepsis obviously upregulated the expression of Jun in hippocampus tissue, whereas the increasing tendency suffered a reversal after CXCL2 knockdown. Additionally, *in vitro* rmCXCL2 stimulation effectively induced the expression of Jun in HT22 neuron. Importantly, when Jun siRNA was transfected into HT22 hippocampal neurons to downregulate the expression of Jun, we observed that CXCL2-induced neuronal ferroptosis was effectively reversed. These findings indicated that microglia-derived CXCL2 induces the neuronal ferroptosis via targeting Jun.

## Conclusions

**Figure 8:**
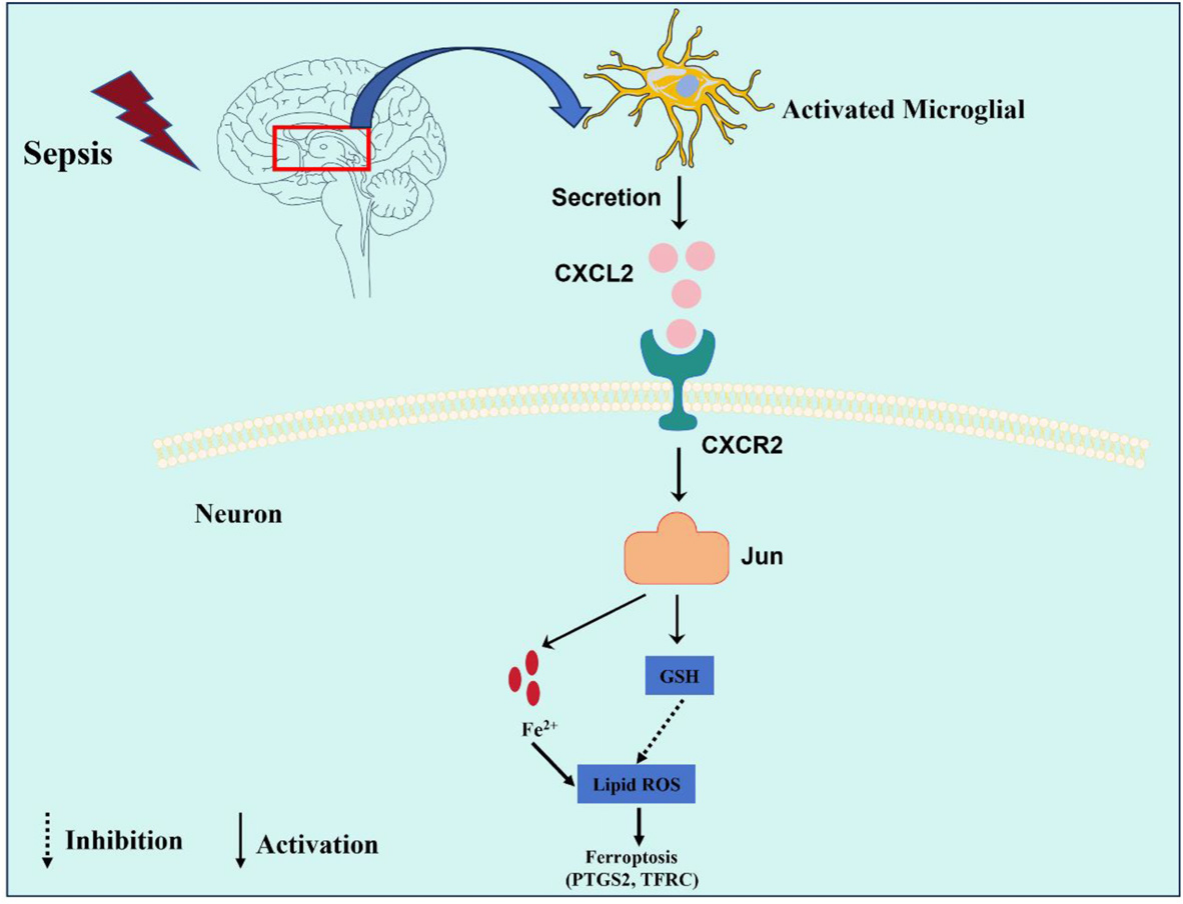
This graphic illustrating the role of the CXCL2/CXCR2/Jun signaling pathway in microglia-neuron crosstalk in SAE. Sepsis promoted the activation of microglia, which expresses and secreted a large amount of CXCL2. CXCL2 induced neuronal ferroptosis by activating the CXCR2/Jun signaling axis of hippocampus neurons, thus aggravates cognitive impairment in SAE.

Our study revealed that microglia-derived CXCL2 aggravated SAE progression by promoting neuronal ferroptosis via Jun, indicating a novel microglia-neuron circuit mediated by CXCL2/CXCR2/Jun signaling in SAE (**Figure 8**).

## Data Availability

The raw sequence data reported in this paper have been deposited in the Genome Sequence Archive (Genomics, Proteomics & Bioinformatics 2021) in National Genomics Data Center (Nucleic Acids Res 2022), China National Center for Bioinformation / Beijing Institute of Genomics, Chinese Academy of Sciences (GSA: CRA023452) that are publicly accessible at https://ngdc.cncb.ac.cn/gsa.
