# Supplementary material for: Microglia-derived CXCL2 induced neuronal ferroptosis via CXCR2/Jun axis in sepsis-associated encephalopathy
Source: Front Immunol. 2025 Mar 6;15:1512300. doi: 10.3389/fimmu.2024.1512300 (PMC11922731; doi:10.3389/fimmu.2024.1512300)
Supplement: Supplementary file 1 [file DataSheet1.zip › Raw Data/original gels.docx]

**Fig 3 Original gels**


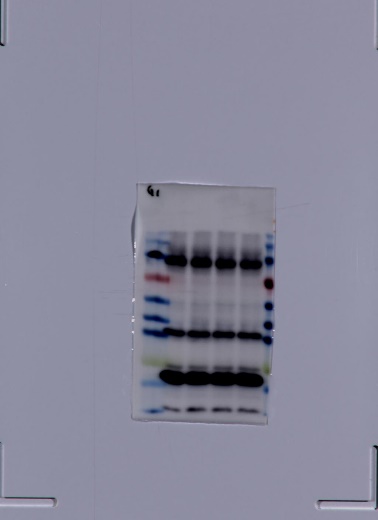

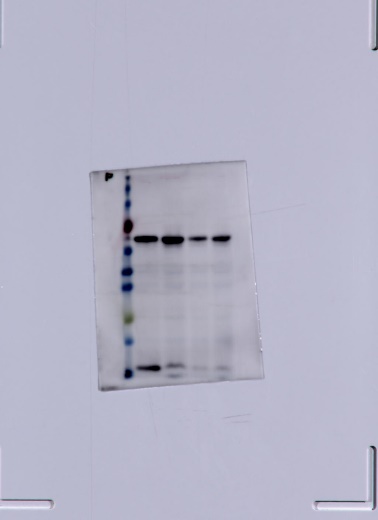

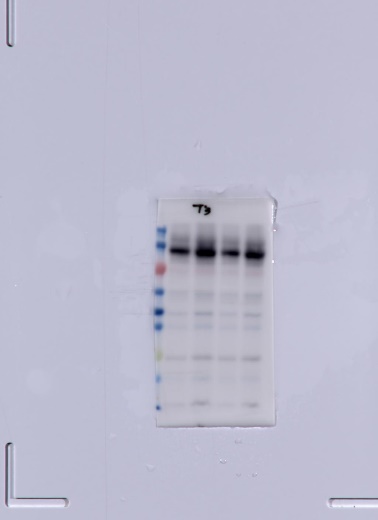


TFRC

PTGS2

GAPDH

**Fig 4 Original gels**


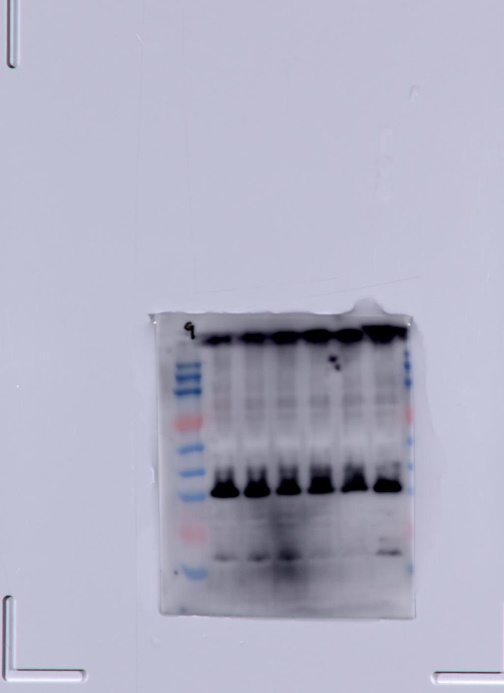

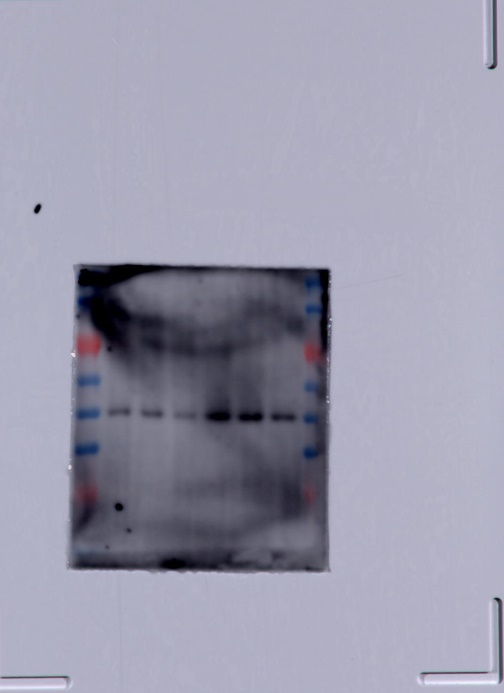


JUN

GAPDH

**Fig 5 Original gels**


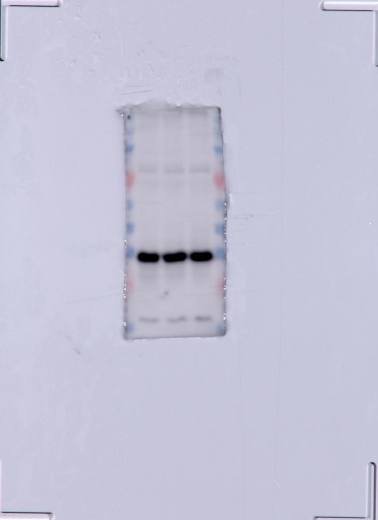

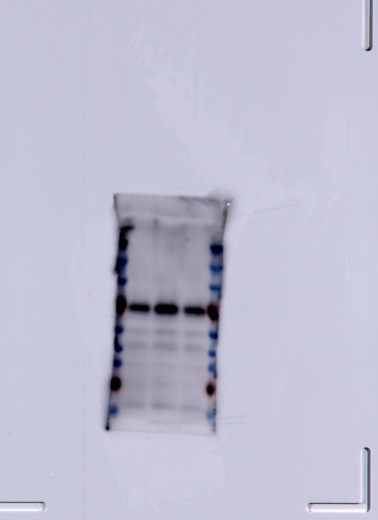

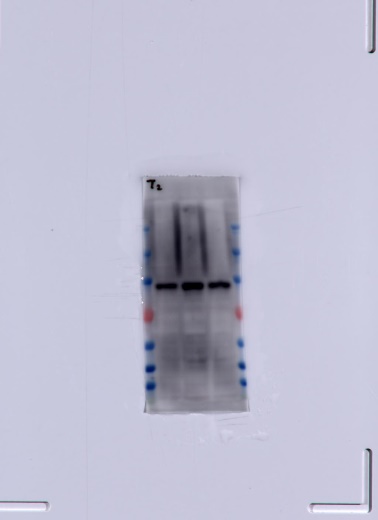


TFRC

PTGS2

GAPDH

**Fig 6 Original gels**


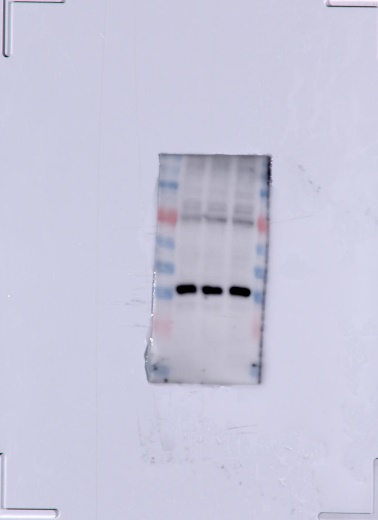

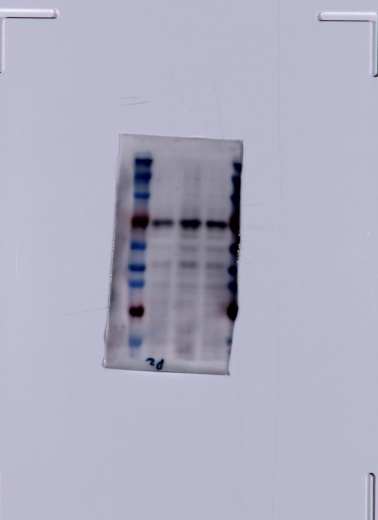

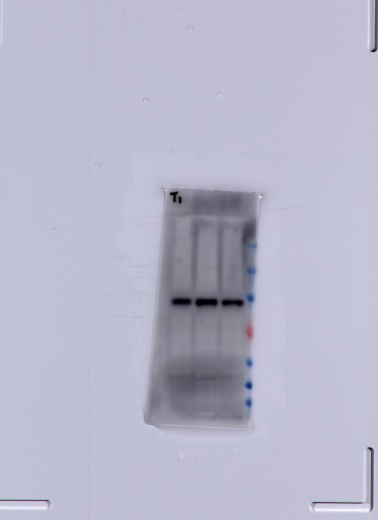


TFRC

PTGS2

GAPDH

**Fig 7 Original gels**


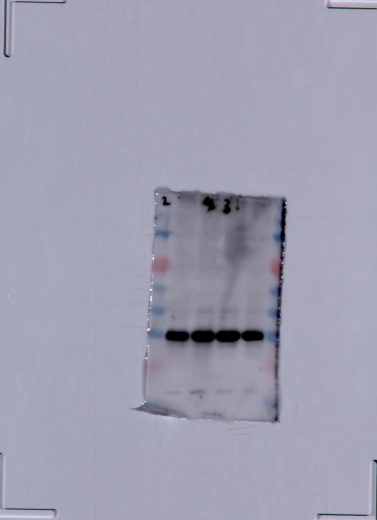

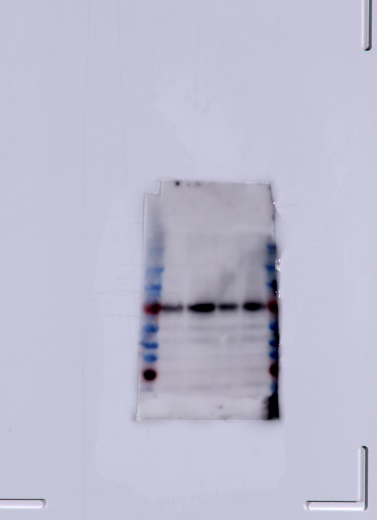

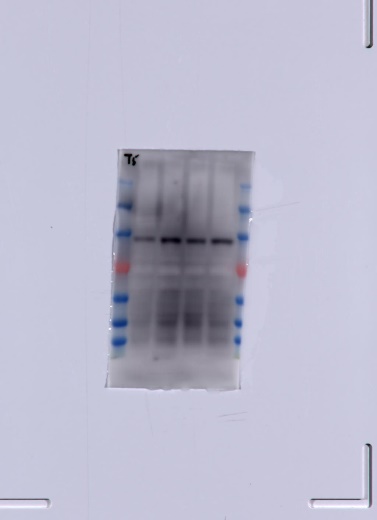


TFRC

PTGS2

GAPDH

**Supplementary Fig 1 Original gels**


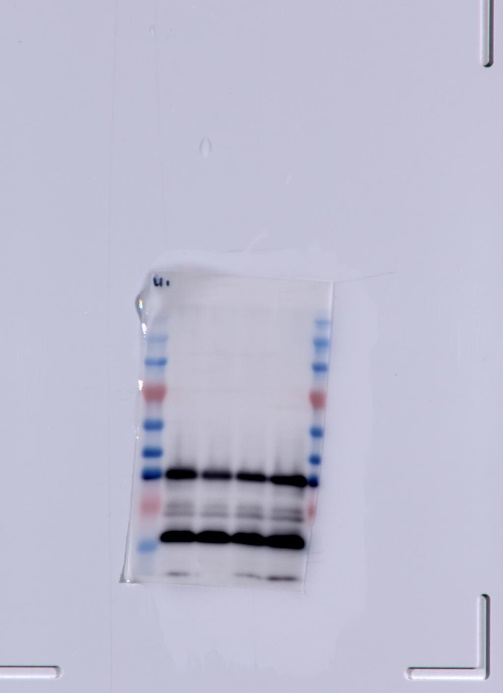

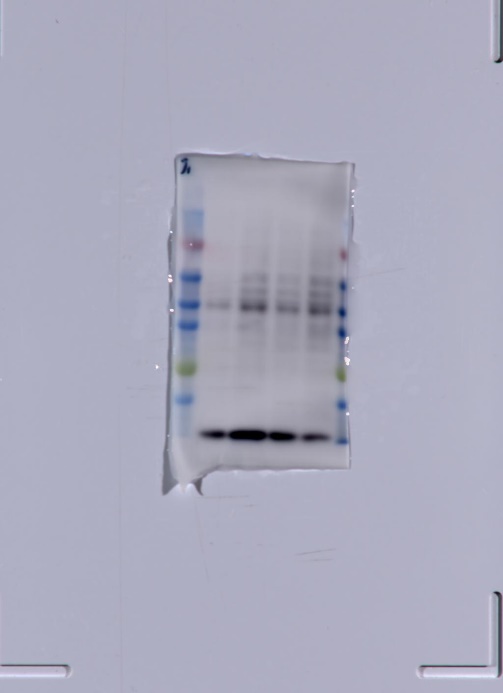


Jun

GAPDH
