# Supplementary figures and images for: Microglia-derived CXCL2 induced neuronal ferroptosis via CXCR2/Jun axis in sepsis-associated encephalopathy
Source: Front Immunol. 2025 Mar 6;15:1512300. doi: 10.3389/fimmu.2024.1512300 (PMC11922731; doi:10.3389/fimmu.2024.1512300)

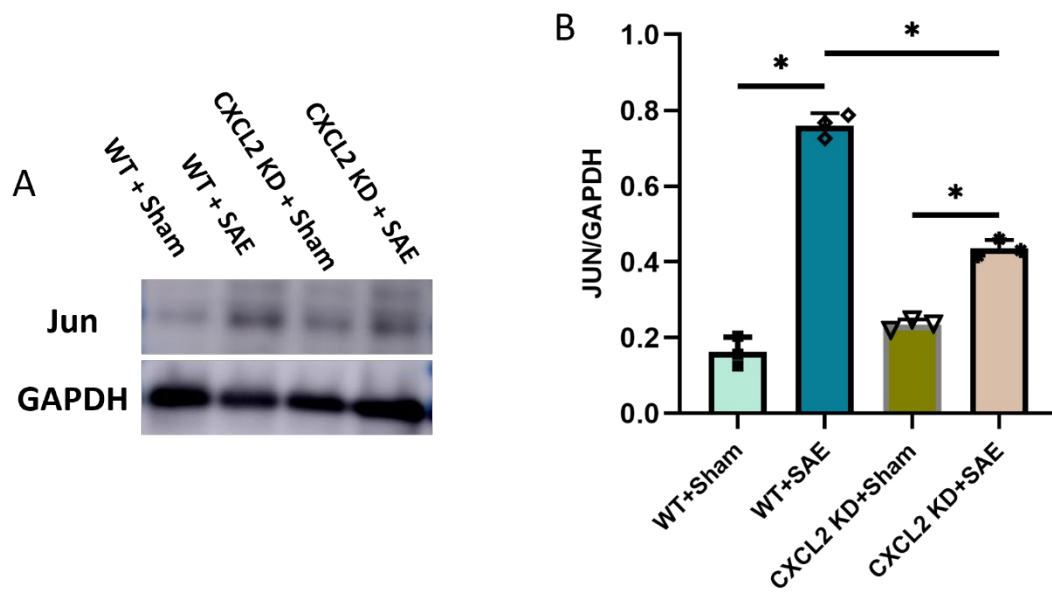

Supplementary Fig 1 The expression of Jun in hippocampus tissue of SAE mice. n=3, \*p<0.05.

Supplement: Supplementary file 2 [file Image1.pdf]
