# Supplementary material for: Microglia-derived CXCL2 induced neuronal ferroptosis via CXCR2/Jun axis in sepsis-associated encephalopathy
Source: Front Immunol. 2025 Mar 6;15:1512300. doi: 10.3389/fimmu.2024.1512300 (PMC11922731; doi:10.3389/fimmu.2024.1512300)
Supplement: Supplementary file 3 [file Table1.pdf]

Supplementary Table 1 mRNA-specific primers of genes

Gene	Primer	Sequence (5'-3')	prodSize
GAPDH	FORWARD	GGCAAATTCAACGGCACAGTCAAG	81bp
	REVERSE	TCGCTCCTGGAAGATGGTGATGG	
CXCR2	FORWARD	TTCTGCTACGGGTTCACACTGC	94bp
	REVERSE	AGACAAGGACGACAGCGAAGATG	
CXCL2	FORWARD	CCACCAACCACCAGGCTACAG	72bp
	REVERSE	TTGGCAGGGTCTTCAGGCATTG	
PTGS2	FORWARD	CTGGTGCCTGGTCTGATGATGTATG	83bp
	REVERSE	GGATGCTCCTGCTTGAGTATGTCG	
TFRC	FORWARD	CGTGGAGACTACTTCCGTGCTAC	145bp
	REVERSE	CTCTTGGAGATACATAGGGCGACAG	
Jun	FORWARD	CAGCCGCCGCACCACTTG	145bp
	REVERSE	TGATCCGCTCCTGAGACTCCATG	
